# Relationship between Exhaled Aerosol and Carbon Dioxide Emission Across Respiratory Activities

**DOI:** 10.1021/acs.est.4c01717

**Published:** 2024-08-13

**Authors:** Benjamin Moseley, Justice Archer, Christopher M. Orton, Henry E. Symons, Natalie A. Watson, Brian Saccente-Kennedy, Keir E. J. Philip, James H. Hull, Declan Costello, James D. Calder, Pallav L. Shah, Bryan R. Bzdek, Jonathan P. Reid

**Affiliations:** † Department of Respiratory Medicine, Royal Brompton Hospital, London SW3 6NP, U.K.; ‡ School of Chemistry, University of Bristol, Bristol BS8 1TS, U.K.; § Department of Respiratory Medicine, Chelsea & Westminster Hospital, London SW10 9NH, U.K.; ∥ National Heart and Lung Institute, Guy Scadding Building, Imperial College London, London SW3 6LY, U.K.; ⊥ Department of Ear, Nose and Throat Surgery, Guy’s & St. Thomas NHS Foundation Trust, London SE1 9RT, U.K.; # Department of Speech and Language Therapy (ENT), Royal National Ear, Nose and Throat and Eastman Dental Hospitals, University College London Hospitals NHS Foundation Trust, London WC1E 6DG, U.K.; ¶ Institute of Sport, Exercise and Health (ISEH), UCL, London W1T 7HA, U.K.; ∇ Ear, Nose and Throat Department, Wexham Park Hospital, Slough SL2 4HL, U.K.; ○ Department of Bioengineering, Imperial College London, London SW7 2AZ, U.K.; ⧫ Fortius Clinic, London W1H 6EQ, U.K.

**Keywords:** carbon dioxide, COVID-19, disease transmission, indoor air aerosol, respiratory aerosol, ventilation

## Abstract

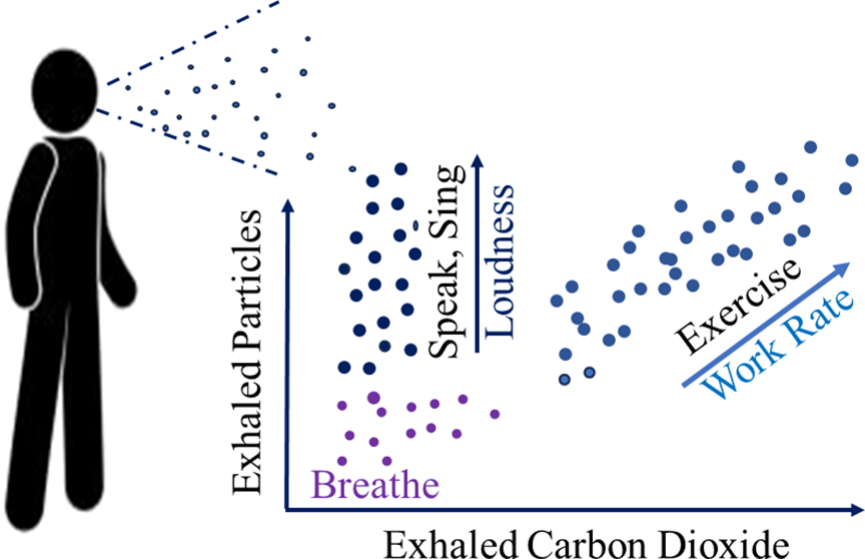

Respiratory particles produced during vocalized and nonvocalized activities such as breathing, speaking, and singing serve as a major route for respiratory pathogen transmission. This work reports concomitant measurements of exhaled carbon dioxide volume (VCO_2_) and minute ventilation (VE), along with exhaled respiratory particles during breathing, exercising, speaking, and singing. Exhaled CO_2_ and VE measured across healthy adult participants follow a similar trend to particle number concentration during the nonvocalized exercise activities (breathing at rest, vigorous exercise, and very vigorous exercise). Exhaled CO_2_ is strongly correlated with mean particle number (*r* = 0.81) and mass (*r* = 0.84) emission rates for the nonvocalized exercise activities. However, exhaled CO_2_ is poorly correlated with mean particle number (*r* = 0.34) and mass (*r* = 0.12) emission rates during activities requiring vocalization. These results demonstrate that in most real-world environments vocalization loudness is the main factor controlling respiratory particle emission and exhaled CO_2_ is a poor surrogate measure for estimating particle emission during vocalization. Although measurements of indoor CO_2_ concentrations provide valuable information about room ventilation, such measurements are poor indicators of respiratory particle concentrations and may significantly underestimate respiratory particle concentrations and disease transmission risk.

## Abstract 2

Respiratory aerosol emissions are correlated with carbon dioxide emission during breathing but not vocalization, which impacts on the application of low-cost carbon dioxide monitors to infer disease transmission risk.

## 1 Introduction

Severe acute respiratory syndrome coronavirus 2 (SARS-CoV-2) causes coronavirus disease 2019 (COVID-19), with severity ranging from asymptomatic to fulminant multiorgan failure and death. Throughout the course of the pandemic, regional prevalence varied through local factors including population demographics, seasonality, and the emergence of differing viral variants. Efforts by healthcare providers and governments to reduce transmission risks centered on reducing social mixing, large scale testing, and the isolation of infected individuals.^1^ Nonpharmaceutical interventions like face masks, physical distancing, hand washing, and surface cleaning were implemented to help slow viral transmission and reduce the burden on healthcare facilities.^2^

The identification of effective mitigation measures is dependent on understanding how a pathogen is transmitted. An important route for human-to-human transmission of SARS-CoV-2 is through the inhalation of respiratory particles or sprayed droplets from an infected person that fall on the mucous membrane of susceptible individuals.^3−6^ Once airborne, SARS-CoV-2 can remain infectious for many minutes or hours depending on local environmental conditions.^7,8^ Studies have reported sometimes conflicting results in size-resolved measurements of the viral load in particles smaller and larger than 5 μm as well as in comparisons of the amount of virus exhaled while speaking, singing, and breathing.^9−12^

There is a continuum of respiratory particles capable of floating in air up to diameters of 100 μm and beyond.^13^ However, three distinct aerosol particle size modes, each able to carry respiratory pathogens, are generated by different mechanisms in the respiratory tract.^14,15^ The smallest particles are created in the small airways of the lower respiratory tract (bronchial mode) during inhalation by film bursting. Aerosol particles of a similar size and larger are also created in the larynx (laryngeal mode) from vibration of the vocal folds during vocalization. The largest particles are created in the upper respiratory tract, including the oral cavity, during oral articulation. Bronchial and laryngeal mode particles are <10 μm diameter and may carry as much as 85% of the viral load of SARS-CoV-2.^12^ Oral mode particles are primarily >20 μm diameter. Oral mode droplets >100 μm diameter have semiballistic trajectories and increased fluid content and can transmit SARS-CoV-2 at close range (<1–2 m).^3^

Exhaled particle number and mass concentrations have been measured and reported as averages over time, with dependencies on expiratory activity (i.e., breathing, speaking, singing), loudness, and participant age.^16−21^ Recently, we have made concurrent measurements of particle size distribution and number concentration with ventilatory parameters to estimate absolute particle emission rates.^17,22^ Such measurements are time-consuming and require costly equipment and specific expertise to operate the equipment and interpret the data, making real world monitoring impractical for the purposes of risk management and minimization.^23^ Combined measurements of carbon dioxide (CO_2_) and vocalization loudness have been proposed as a surrogate measure for assessing inhalation-based infection risk.^24^

Ambient CO_2_ levels are typically ∼0.04% by volume (∼400 ppm) of air at standard atmospheric pressure and temperature. In a plume of exhaled air, CO_2_ concentrations are orders of magnitude higher, around 4% by volume (40,000 ppm). Therefore, CO_2_ detectors have been proposed as sensitive, cheap, and fast approaches to identify areas of inadequate ventilation in any indoor space and, thus, enhanced risk of respiratory pathogen transmission by inhalation.^25−27^ Aerosol particles <5 μm can be expected to largely follow the initial exhalatory jet before being carried by convection air currents, with displacement rates in a room similar to that measured for CO2 gas,^28,29^ although particles >1 μm are also significantly impacted by gravitational settling.^30^

In this study, we quantify the absolute amounts of aerosol in the bronchial and laryngeal modes (both ≤5 μm) along with the exhaled volume of CO_2_ using a novel methodology based on source-specific respiratory measurement. We report concomitant measurements of the volume of produced carbon dioxide (VCO_2_) and minute ventilation (VE), along with particle number and mass concentrations and emission rates, enabling exploration of relationships among these parameters during vocalized and nonvocalized activities.

## 2 Study Protocols and Methods

### 2.1 Human Participants

This study was approved by the Public Health England Research Ethics and Governance of Public Health Practice Group (PHE REGG, NR0221) within the PERFORM-2 project. 33 healthy adult participants were recruited (age: 29 to 63 years; gender: 17 males and 16 females; normal body mass index (BMI) at 23.8 kg m^–2^, SD ± 4.1) with no significant respiratory or cardiovascular illness.^22^ Of the 33 participants, 25 (spanning a range of athletic capabilities) completed cardio-pulmonary exercise testing (CPET), with a mean peak oxygen uptake per kg body mass (VO_2_ kg^–1^) of 42.4 mL kg^–1^ min^–1^ (SD ± 11.01, range 26 to 65). The remaining eight adults were a subset of professional singers from our previous studies.^16,17^ All participants were prescreened to ensure they had no COVID-19 symptoms, tested negative for COVID-19 via lateral flow, and refrained from smoking, vigorous exercising, consuming alcohol, or eating heavily for 4 h prior to taking part in the experiment. Written informed consent was documented from all participants.

### 2.2 Experimental Protocols

This study follows the methods used in our previous work examining aerosol emission rates during exercise^22^ and singing,^17^ where both minute ventilation and aerosol emission rates were measured. Twenty-five adult participants chosen for the exercise activities completed a maximal 30, 40, or 50 W ramp CPET protocol to voluntary exhaustion on a cycle-ergometer [CORTEX MetaLyzer 3B-R3 + Wattbike Atom (Next Generation) cycle ergometer or Vyaire Medical Vyntus CPX + VIAsprint 200P W/BP Serial Ergometer system]. This procedure was consistent with CPET international guidelines to characterize exercise capacity and ventilatory response.^35^ CPET data were recorded and analyzed using Cortex MetaSoft Studio Version 5.12.0 (Cortex system) and SentrySuite software V. 320 (Vyaire Medical system).

After the maximal ramp CPET protocol, participants were instructed to rest for at least 1 h and then to complete a second stepped exercise test where exercise intensities were established from the previous maximal CPET. The two exercise intensities completed were vigorous (80% of the participants anaerobic threshold (AT)) and very vigorous (AT + 30% Δwork rate (WR)). Work-rates were assigned using a BORG CR10 scale. The second stepped activities involved each participant breathing at rest for 60 s, followed by speaking the “Happy Birthday” song to “Susan” for 60 s at 70–80 dBA.^16,17,22^ Participants then completed vigorous (∼6 min) and very vigorous (∼4 min) exercise. Minute ventilation, exhaled CO_2_, and aerosol measurements were taken after 2 min of vigorous exercise and 30 s of very vigorous exercise.^22,31^

The separate group of eight adult professional singers performed a series of vocalization activities including speaking at 70–80 dBA, singing at 70–80 dBA, and singing at 90–100 dBA using the “Happy Birthday” song addressing “Susan”. Each activity lasted for 20 s followed by 30 s rest.^16,17^

### 2.3 Measurements of Aerosol and Respiratory Parameters

Minute ventilation (VE) and exhaled CO_2_ (VCO_2_) were recorded using a modified Hans Rudolph 7450 series V2 mask. Respiratory particle number concentrations were measured using an aerodynamic particle sizer (APS; TSI Inc. model 3321; 1 L min^–1^ sample flow rate, 4 L min^–1^ sheath flow rate, size range 0.54–20 μm aerodynamic diameter). Aerosols were sampled from a modified CPET mask with a 6 mm sampling port cut at the tip of the nose to allow attachment of the sampling tube. The sampling port location was chosen to reduce the risk of collected water droplets pooling in the facemask and interfering with the measurement.^22^ Similarly, minute ventilation and respired aerosol particles were measured from the subset of 8 adult professional singers using a noninvasive Vyntus Hans Rudolf mask, housing a rotating vane spirometer and connected to an APS.^17^ Vocalization sound pressure levels were recorded simultaneously with 1 s sampling intervals for both speaking and singing activities.

Both the exercise and vocalization measurements were carried out in a laminar flow operating theater with sufficient air changes per hour to ensure participants inhaled particle-free air in the 0.54–20 μm diameter size range. Consequently, particles detected by the APS could be confidently attributed to the participants’ expiratory activities, with the particle number concentration returning to 0 cm^–3^ during sampling pauses.^16^ Room temperature and relative humidity were controlled at approximately 18 °C and 40% RH, respectively.

### 2.4 Data Processing and Statistical Analysis

The raw data of particle counts from the APS instrument were collected using the Aerosol Instrument Manager software package (TSI, USA) and postprocessed with custom-written software in LabVIEW. The postprocessed files were then analyzed in Origin (OriginLab). For the statistical analysis, we used a similar approach to our previous work.^16,17,19,22^ Data were inspected, and log transforms were utilized when the data were skewed. For pairwise comparisons between activities, independent sample *t*-tests were used whereas for comparisons of different activities within individuals, paired *t*-tests were employed.

## 3 Results

### 3.1 VE, Exhaled Carbon Dioxide, and Particle Number Concentrations

**Figure 1:**
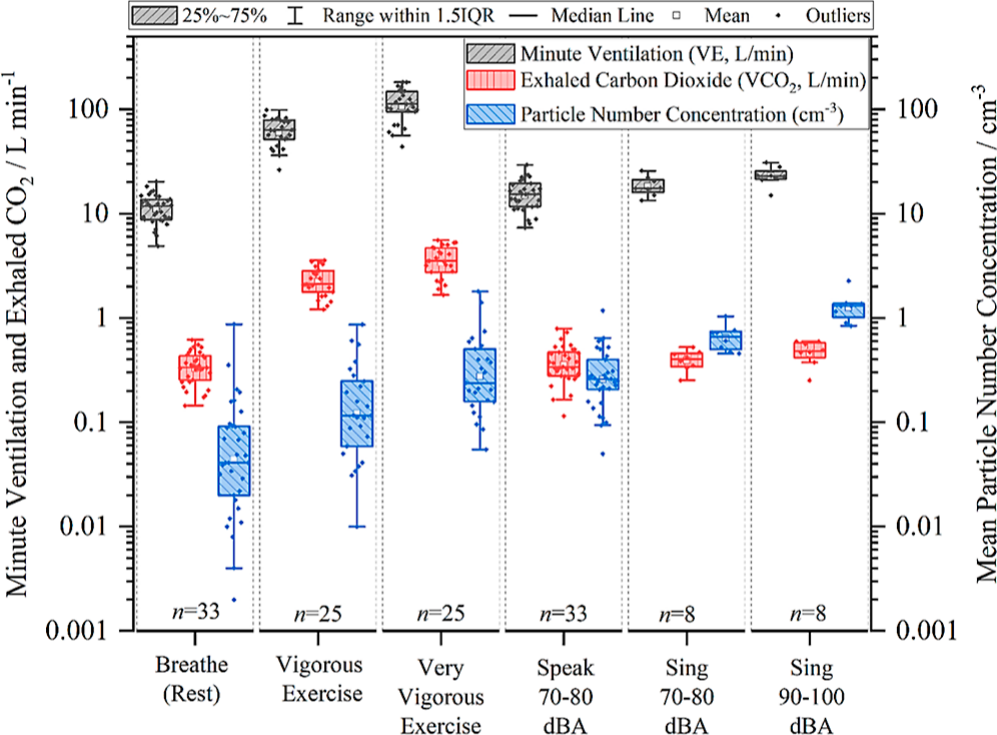
Mean particle number concentration (blue), minute ventilation (VE, gray), and exhaled carbon dioxide (VCO_2_, red) for the same series of activities. Boxes show the mean, median, and interquartile range (IQR). Whiskers indicate the range (data within 1.5 IQR).

Figure 1 shows VE (L min^–1^), VCO_2_ (L min^–1^), and particle number concentration (cm^–3^) measured across the 33 adult participants during exercise and vocalization maneuvers. Corresponding numerical values are provided in Table S1. VE and VCO_2_ quantify the mean volume of air and CO_2_, respectively, expelled from the participant in 1 min of an activity at standard temperature and pressure. The particle number concentration is for particles in the 0.54–20 μm aerodynamic diameter size range.

For nonvocalized exercise activities, VE and VCO_2_ follow a similar trend to particle number concentration. In terms of median values, VE measurements for vigorous and very vigorous exercise were 5 and 10 times greater than for breathing at rest, respectively (*p* < 0.001 for both). Vigorous and very vigorous exercise also generated six and ten times more VCO_2_, respectively, than breathing at rest (*p* < 0.001 for both). With respect to particle number concentrations, vigorous and very vigorous exercise generated three and six times more particles (in terms of median values) than breathing at rest (*p* < 0.001 for both).

When vocalizing, VE measurements during speaking at 70–80 dBA are comparable to breathing at rest (*p* = 0.15), whereas VE measurements during singing at 70–80 dBA (*p* = 0.002) and singing at 90–100 dBA (*p* < 0.001) are modestly (but significantly) different from breathing. VCO_2_ during speaking at 70–80 dBA (*p* = 0.268) and singing at 70–80 dBA (*p* = 0.226) are not different to that emitted during breathing at rest. Both speaking at 70–80 dBA (*p* = 0.104) and singing at 70–80 dBA (*p* = 0.215) had comparable VCO_2_ to singing at 90–100 dBA. However, VCO_2_ during breathing at rest is modestly lower (∼0.7×) than that during singing at 90–100 dBA (*p* = 0.020). These differences may arise from a lack of statistical power owing to the small cohort size for professional singers (*n* = 8). With respect to particle number concentrations, speaking at 70–80 dBA, singing at 70–80 dBA, and singing at 90–100 dBA all generate significantly more particles than breathing (*p* < 0.001). Singing at 90–100 dBA generates five times and two times more aerosol particles than speaking at 70–80 dBA (*p* < 0.001) and singing at 70–80 dBA (*p* < 0.001), respectively, as well as 32 times more particles than breathing at rest (*p* < 0.001). This observation confirms earlier results concluding that a vocalization’s loudness is a key factor governing respiratory particle concentrations generated by expiratory maneuvers.^16,21,28^

### 3.2 Comparison of Absolute Particle Emission Rates to Exhaled Carbon Dioxide

Minute ventilation measurements allow estimation of both the absolute number of particles and the CO_2_ concentration carried in the exhaled air. The absolute number emission rate (eq 1) accounts for activity-specific changes in both minute ventilation and number concentration. The CO_2_ concentration (in ppm) in an indoor space can be used as an indication of the potential risk of transmission,^25−27^ and the CO_2_ concentration in the respiratory plume can be estimated from eq 2. Figures S1 and S2 report the CO_2_ concentration in exhaled air (in ppm) and particle number emission rates (s^–1^), respectively, with numerical values provided in Table S2.
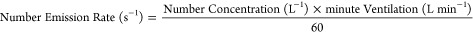
1
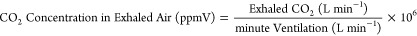
2

Another informative representation of the measured aerosol data is particle mass, which is inferred from the size-resolved particle number concentration measurements. Figure S3 shows mean particle mass concentrations (assuming a particle density equal to that of water, 1 g cm^–3^) during exercising and vocalizing. The median mass concentrations exhaled during vigorous (0.17 μg m^–3^, IQR 0.07–0.34) and very vigorous (0.42 μg m^–3^, IQR 0.24–0.66) exercise are 7.3 and 18 times higher than those exhaled during breathing (*p* < 0.001 for both), respectively (see Table S2). For activities involving vocalization, speaking and singing generate 17- and 51-times higher mass concentrations at 70–80 dBA than breathing at rest (*p* < 0.001 for both). Singing at 90–100 dBA results in a median particle mass concentration value of 2.9 μg m^–3^ (IQR 2.0–4.6), that is 7.3- and 2.3-fold higher than speaking at 70–80 dBA (*p* < 0.001) and singing at 70–80 dBA (*p* = 0.001), respectively, and 120 times higher than breathing at rest (*p* < 0.001). Combining the particle mass concentration estimates with minute ventilation enables estimation of the absolute particle mass emission rate (see Figure S4) during a respiratory activity (17, 22).

**Figure 2:**
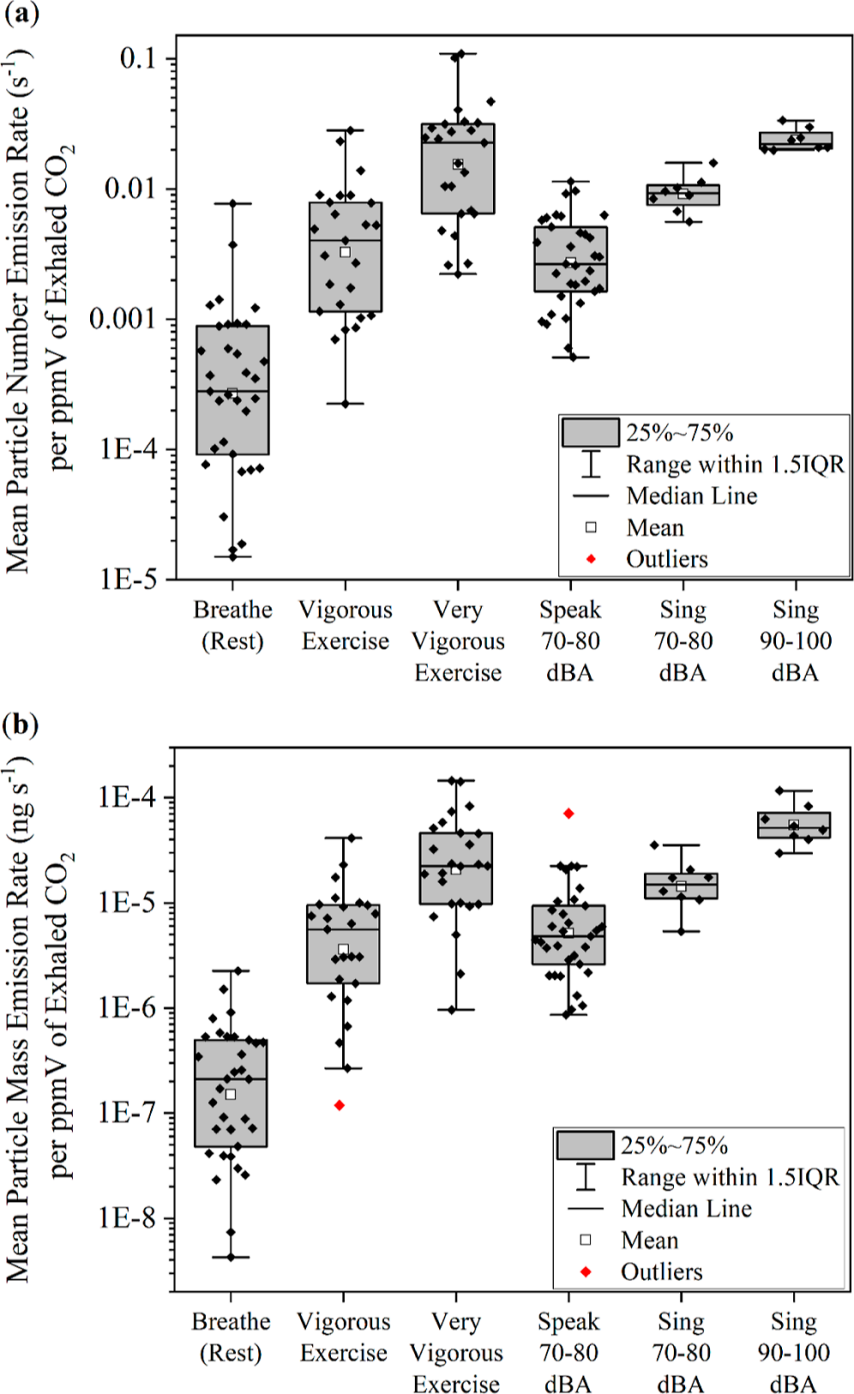
Mean particle (a) number and (b) mass emission rates per ppm of exhaled CO_2_ for a range of nonvocalized and vocalized activities.

To investigate whether CO_2_ levels are a useful proxy for respiratory particle concentration, we first examine particle number and mass emission rates divided by the CO_2_ concentration in ppm. The goal of this comparison is to explore whether an increase in measured CO_2_ concentration (typically reported in ppm by CO_2_ monitors) corresponds to a quantifiable increase in particle emission across the full range of studied respiratory activities. Figure 2 plots mean particle number (Figure 2a) and mass (Figure 2b) emission rates per ppm of exhaled CO_2_ during exercising and vocalizing. Mean particle number and mass emission rates generated per ppm of exhaled CO_2_ increase significantly as work-rates of exercise activity increase despite the decrease in exhaled CO_2_ (in ppm) during very vigorous exercise (see Figure S1). Vigorous and very vigorous exercise lead to a 14- and 81-fold increase in number emission rate and a 26- and 107-fold increase in mass emission rate, respectively, per ppm of exhaled CO_2_ compared to breathing at rest (*p* < 0.001). Differences in the number and mass emission rates generated per ppm of exhaled CO_2_ during very vigorous exercise are also significant compared to vigorous exercise (*p* < 0.001).

When vocalizing, mean number and mass emission rates generated per ppm of exhaled CO_2_ also increase as voice loudness increases despite the modest decrease in exhaled CO_2_ (in ppm) as voice loudness increases. Singing at 90–100 dBA results in an 8-fold increase in number and an 11-fold increase in mass emission rates per ppm of exhaled CO_2_ compared to speaking at 70–80 dBA (*p* < 0.001 for both). Meanwhile, singing at 90–100 dBA results in a 2-fold increase in number and 3-fold increase in mass emission rates per ppm of exhaled CO_2_ compared to singing at 70–80 dBA (*p* < 0.001 for both). Speaking and singing at 70–80 dBA as well as singing at 90–100 dBA have significantly higher number and mass emission rates per ppm of exhaled CO_2_ when compared to breathing at rest (*p* < 0.001 for both). In short, particle emission rates are decoupled from the emitted CO_2_ concentration.

**Figure 3:**
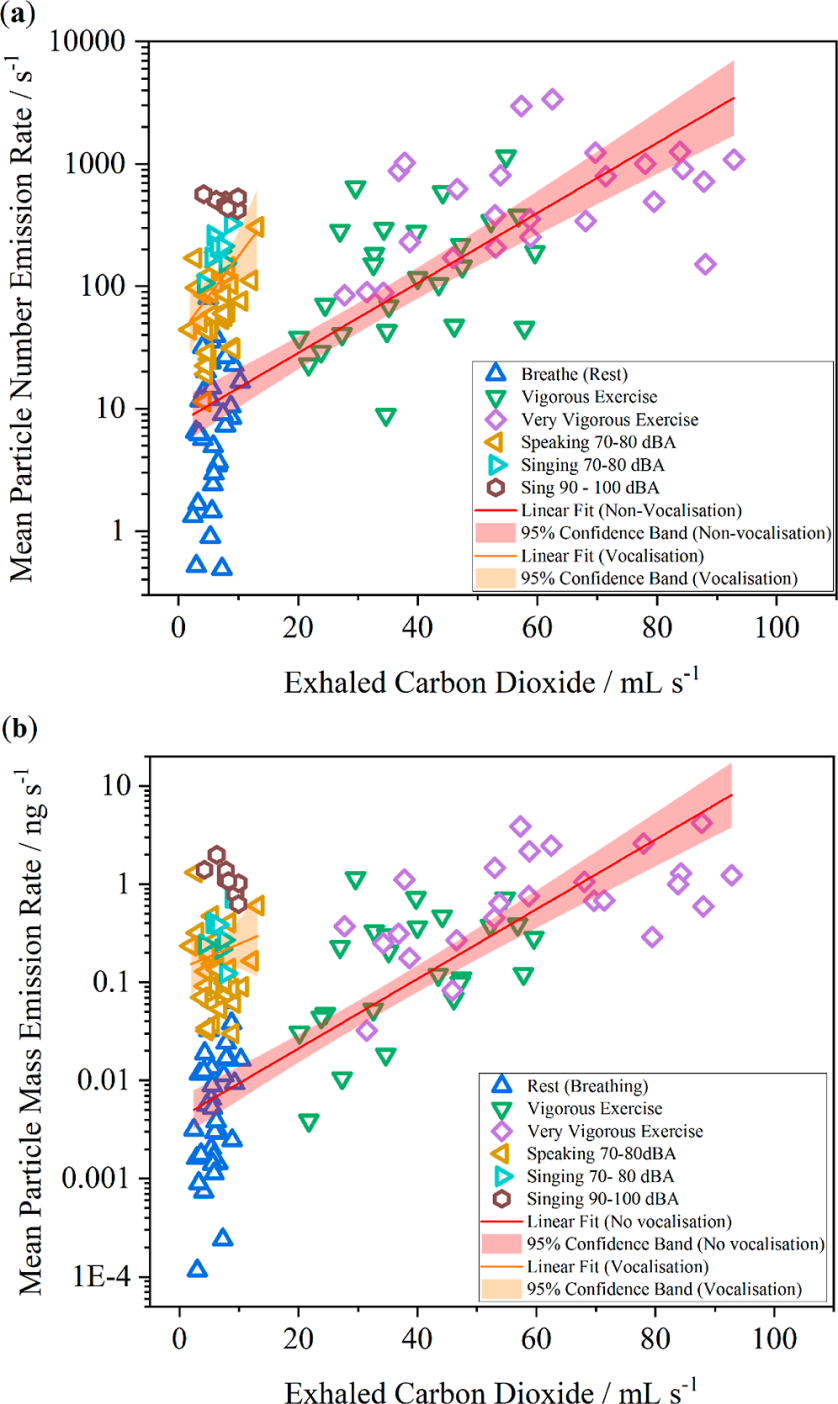
Plots of (a) mean particle number and (b) mean particle mass emission rate per exhaled CO_2_ (mL s^–1^) for activities not requiring vocalization (i.e., breathing at rest, vigorous exercise, and very vigorous exercise) and activities involving vocalization (speaking at 70–80 dBA, singing at 70–80 dBA, and singing at 90–100 dBA).

Another way to explore a possible relationship between particle and CO_2_ emission is to plot particle emission rates against the directly measured CO_2_ emission rate (expressed in mL of gas exhaled in 1 s at standard temperature and pressure, i.e., mL s^–1^). Figure 3 reports mean particle number (Figure 3a, in s^–1^) and mass (Figure 3b, in ng s^–1^) emission rates against CO_2_ emission rates for activities not requiring vocalization (i.e., breathing at rest, vigorous exercise, and very vigorous exercise) and activities involving vocalization (speaking at 70–80 dBA, singing at 70–80 dBA, and singing at 90–100 dBA). A strong correlation between exhaled CO_2_ emission rate and mean particle number (*r* = 0.81) and mass (*r* = 0.84) emission rate is observed for the activities not requiring vocalization. In contrast, the exhaled CO_2_ emission rate is poorly correlated with mean particle number (*r* = 0.34) and mass (*r* = 0.12) emission rates during activities that involve vocalization.

## 4 Discussion

Ambient CO_2_ measurements are emerging as a fast, cheap, and simple approach to identify whether an indoor space is adequately ventilated^32^ and whether the space may pose an enhanced risk of disease transmission through the inhalation route.^25−27^ This study explores the relationship between exhaled CO_2_ volume and respiratory particle emission rate across activities that do and do not require vocalization. The key finding is that a correlation is observed between the exhaled CO_2_ emission rate (mL s^–1^) and respiratory particle number and mass emission rates only for activities not involving vocalization (Figure 3, *r* ≥ 0.8). In contrast, a poor correlation exists when the respiratory activities involve vocalization (*r* < 0.4). The reason for the lack of correlation between particle and CO_2_ emission rates for activities involving vocalization is due to the production of larger particles in the larynx, a defining feature of the vocalization size distribution that is highly sensitive to the loudness of the vocalization.^14,16^ Consequently, the mechanism of CO_2_ generation becomes decoupled from respiratory parameters during vocalization. Therefore, an increase in CO_2_ concentration does not correspond to a consistent and quantifiable increase in respiratory particle emission (Figure 2). Because of this lack of correlation between respiratory particle and CO_2_ emission, using ambient CO_2_ concentrations to assess risk from respiratory particles in indoor environments could lead to significant underestimation of respiratory particle concentration if vocalization (i.e., speaking or singing) is taking place. In this case, CO_2_ concentration measurements remain a useful proxy for assessing the extent of ventilation in an indoor environment but not the amount of circulating aerosol and potential pathogen. This could be amplified by the increased stability of infectious virus in aerosol reported at high CO_2_ concentration.^33^

Even during exercise, our findings suggest some caution should be applied when using CO_2_ concentration to infer respiratory particle emission due to the impact of exercise hyperpnoea. In the near exhaustive state that follows saturation of the homeostatic mechanisms that maintain blood pH, the respiratory compensation point is reached, and ventilation (i.e., VE) increases discordantly from CO_2_ output.^34^ Therefore, CO_2_ concentration measured in ppm may appear to fall despite an increased total ventilatory output (Figure S1), as the CO_2_ emission rate does not increase monotonically with increasing VE (Figure 1).

Our results are broadly consistent with those of Good et al.^24^ (2021), who also measured both respiratory particle and CO_2_ concentrations. They found that singing generated more respiratory particles on a per breath or per-CO_2_ basis than speaking, and that vocalizing at higher loudness was also associated with generation of more particles on a per-CO_2_ basis than at lower loudness. However, our study explores a wider range of activities (including breathing and exercise) and quantifies particle emission rates rather than concentrations.

In conclusion, this study characterizes the relationship between exhaled CO_2_ and respiratory particle generation during exercising and vocalizing. We demonstrate that the CO_2_ emission rate is correlated with the respiratory particle emission rate only during activities not involving vocalization (i.e., exercising). The CO_2_ emission rate is poorly correlated with respiratory particle emission rates during activities requiring vocalization (i.e., speaking or singing). The lack of correlation during vocalization is due to the different mechanism of respiratory particle generation, which is dominated by particle formation in the larynx and is highly sensitive to the loudness of the vocalization. When considering exhaled CO_2_ production as a concentration (ppm), particle emission rates per ppm of CO_2_ emitted vary significantly across activities. In contexts where respiratory particle emission is dominated by breathing (e.g., a gym), CO_2_ concentration measurements could be a reasonable metric for assessing the release of respiratory particles capable of carrying pathogens. However, CO_2_ concentration measurements are very likely to underestimate the contribution of respiratory particles to ambient aerosol concentrations in environments where activities involve vocalization (e.g., social environments). Although CO_2_ measurements are unlikely to provide quantitative estimates of respiratory particle emission, these measurements can still provide valuable information about ventilation in indoor environments.

## Data Availability

Data underlying the figures are publicly available in the BioStudies database (https://www.ebi.ac.uk/biostudies/) under accession S-BSST1431.

## Supporting Information Available

Plots of exhaled CO_2_ (ppmV), mean aerosol particle number emission rates, mean particle mass concentrations, mean particle mass emission rates, and tables providing summaries of minute ventilation, exhaled CO_2_, particle number concentration data (PDF)
