# Supplementary material for: Relationship between Exhaled Aerosol and Carbon Dioxide Emission Across Respiratory Activities
Source: Environ Sci Technol. 2024 Aug 13;58(34):15120–6. doi: 10.1021/acs.est.4c01717 (PMC11360368; doi:10.1021/acs.est.4c01717)
Supplement: Supplementary file 1 — es4c01717_si_001.pdf [file es4c01717_si_001.pdf]

The Relationship Between Exhaled Aerosol and Carbon Dioxide Emission Across Respiratory Activities

Benjamin Moseley[1†], Justice Archer[2†], Christopher M. Orton[1,3,4], Henry E. Symons[2], Natalie A. Watson[5], Brian Saccente-Kennedy[6], Keir E.J. Philip[1,4], James H. Hull[1,7], Declan Costello[8], James D. Calder[9,10], Pallav L. Shah[1,3,4], Bryan R. Bzdek[2*], Jonathan P. Reid[2*]

[1]Department of Respiratory Medicine, Royal Brompton Hospital, London SW3 6NP, UK

[2]School of Chemistry, University of Bristol, Bristol BS8 1TS, United Kingdom

[3]Department of Respiratory Medicine, Chelsea & Westminster Hospital, London SW10 9NH, UK

[4]National Heart and Lung Institute, Guy Scadding Building, Imperial College London, London SW3 6LY, UK

[5]Department of Ear, Nose and Throat Surgery, Guy's & St. Thomas NHS Foundation Trust, London SE1 9RT, UK.

[6]Department of Speech and Language Therapy (ENT), Royal National Ear, Nose and Throat and Eastman Dental Hospitals, University College London Hospitals NHS Foundation Trust, London WC1E 6DG, UK

[7]Institute of Sport, Exercise and Health (ISEH), UCL, London W1T 7HA, UK

[8]Ear, Nose and Throat Department, Wexham Park Hospital, Slough SL2 4HL, UK

[9]Department of Bioengineering, Imperial College London, London SW7 2AZ, UK

[10]Fortius Clinic, London W1H 6EQ, UK

† First authors

*Corresponding authors: b.bzdek@bristol.ac.uk, J.P.Reid@bristol.ac.uk

Supporting Information

7 pages, 4 figures, 2 tables

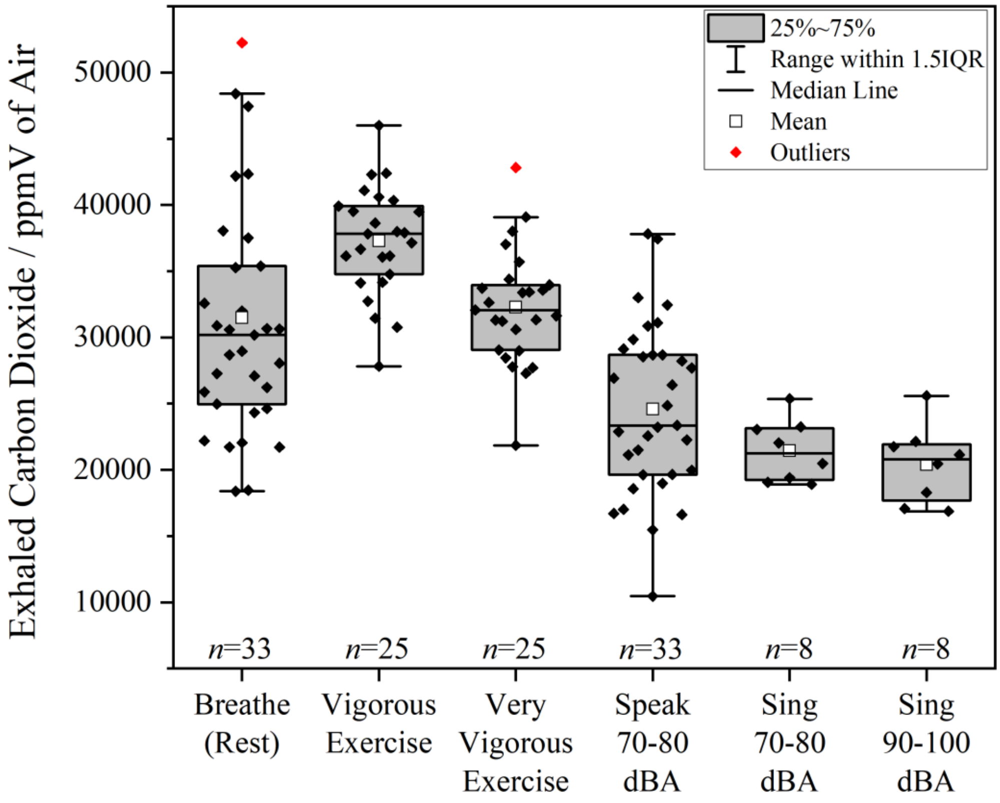

Figure S1: Exhaled CO_2 (ppmV) for activities not requiring vocalization (i.e., breathing at rest, vigorous exercise, and very vigorous exercise) and activities involving vocalization (speaking at 70-80 dBA, singing at 70-80 dBA, and singing at 90-100 dBA). Boxes indicate mean, median, and interquartile range (IQR), whiskers indicate range (data within 1.5 IQR).

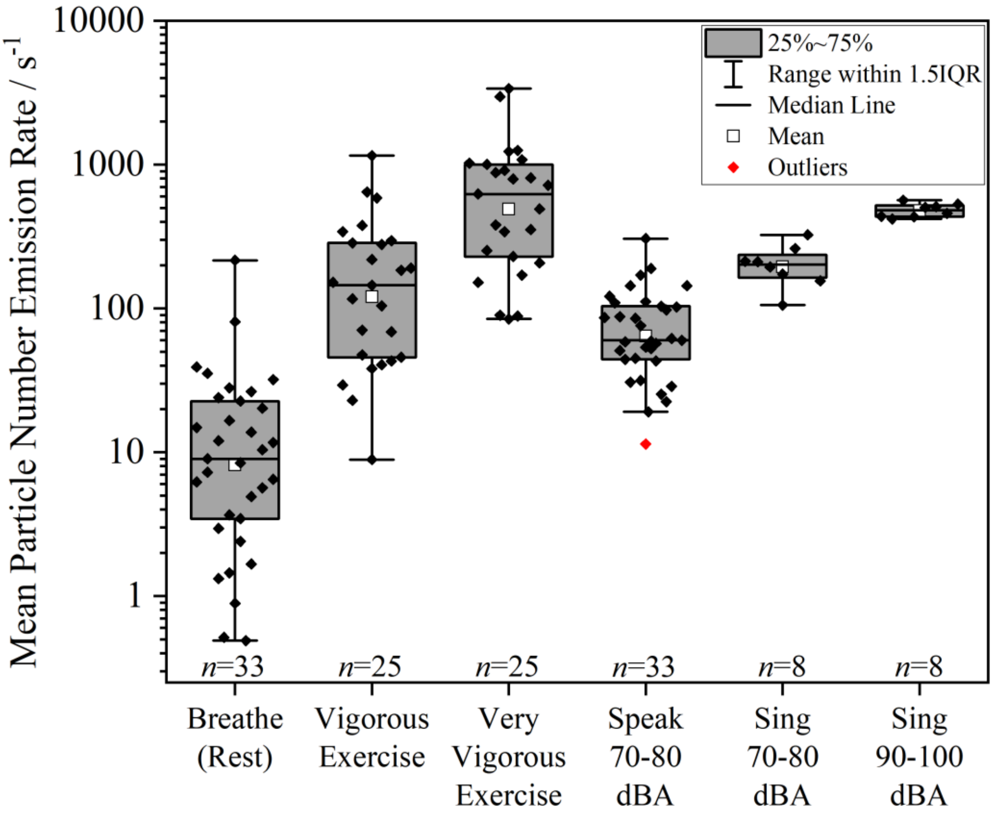

Figure S2: Mean particle number emission rate (s^{-1}) for a range of nonvocalized and vocalized respiratory activities.

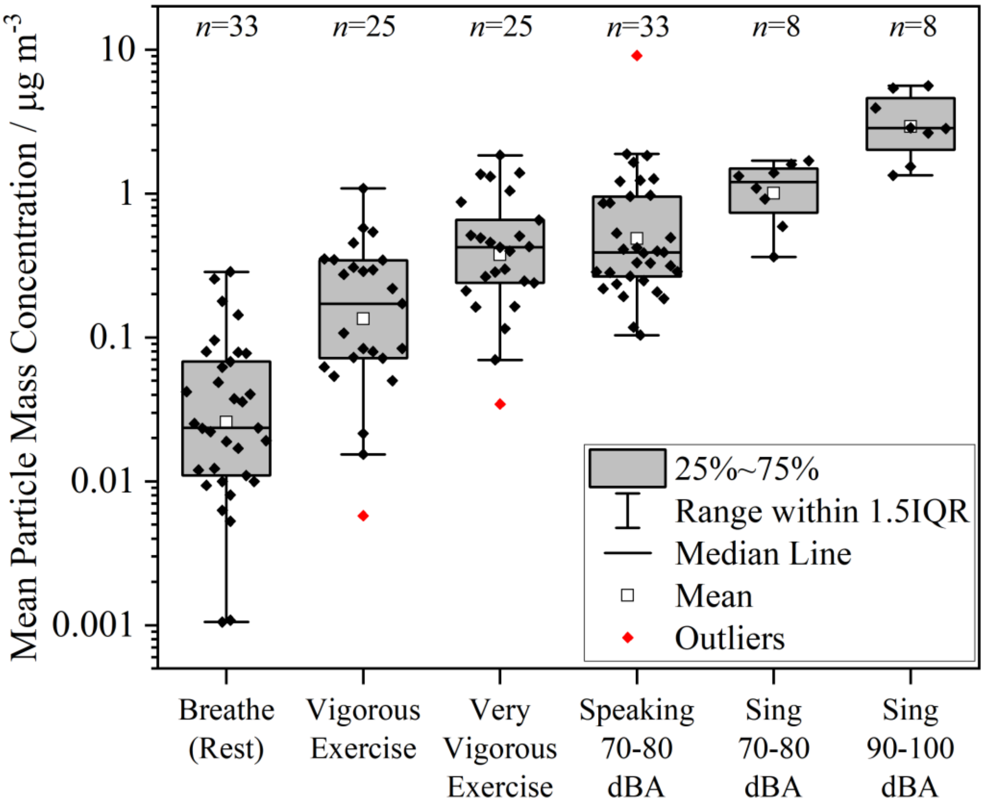

Figure S3: Mean particle mass concentration for a range of nonvocalized and vocalized activities.

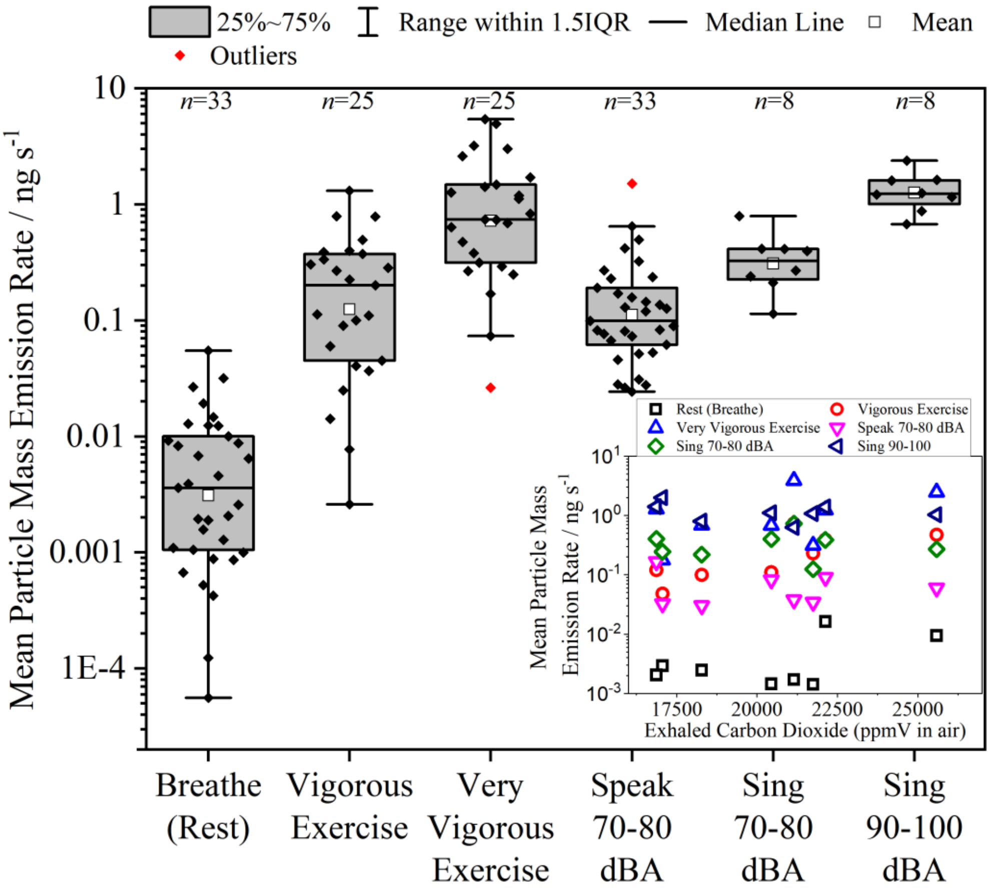

Figure S4: Mean particle mass emission rate (ng s^{-1}) for vocalized and nonvocalized respiratory activities. The inset plots mean particle mass emission rate against the exhaled CO_2 concentration (ppm) in the exhalation jet for all the activities.

Table S1: Summary of minute ventilation, exhaled CO_2, and particle number concentrations reported in Figure 1.

Quantities	Parameters	Activities					
		Breathe (Rest)	Vigorous Exercise	Very Vigorous Exercise	Speak 70-80 dBA	Sing 70-80 dBA	Sing 90-100 dBA
Minute Ventilation (VE, L min^{-1})	Mean	10.9	59.8	106.4	15.0	18.3	22.8
	Median	11.8	63.3	112.3	15.4	17.6	22.9
	25%	8.8	51.8	95.3	11.8	16.1	21.6
	75%	13.7	78.9	147.8	19.5	21.2	25.6
	Bottom Whisker	4.9	26.3	43.8	7.3	13.4	15.0
	Top Whisker	20.3	98.9	182.2	29.4	25.7	30.9
	n	33	25	25	33	8	8
Exhaled Carbon Dioxide (VCO_2, L min^{-1})	Mean	0.33	2.22	3.40	0.36	0.39	0.46
	Median	0.34	2.11	3.53	0.34	0.40	0.48
	25%	0.26	1.78	2.76	0.28	0.34	0.42
	75%	0.44	2.83	4.68	0.47	0.46	0.58
	Bottom Whisker	0.15	1.21	1.67	0.12	0.25	0.25
	Top Whisker	0.62	3.58	5.57	0.79	0.53	0.60
	n	33	25	25	33	8	8
Particle Number Concentration (cm^{-3})	Mean	0.044	0.121	0.277	0.258	0.641	1.26
	Median	0.041	0.116	0.238	0.260	0.660	1.31
	25%	0.020	0.059	0.158	0.207	0.500	1.02
	75%	0.092	0.248	0.504	0.400	0.741	1.38
	Bottom Whisker	0.002	0.01	0.055	0.05	0.457	0.841
	Top Whisker	0.871	0.866	1.80	1.179	1.04	2.27
	n	33	25	25	33	8	8

Table S2: Summary of exhaled carbon dioxide in ppm (Figure S1), particle number emission rate in s^{-1} (Figure S2) aerosol mass concentration in µg m^{-3} (Figure S3), and particle mass emission rate in ng s^{-1} (Figure S4).

Quantities	Parameters	Activities					
		Breathe (Rest)	Vigorous Exercise	Very Vigorous Exercise	Speak 70-80 dBA	Sing 70-80 dBA	Sing 90-100 dBA
Exhaled Carbon Dioxide (ppmV in air)	Mean	30125.7	37059.3	31975.8	23717.9	21330.7	20229.5
	Median	30175.0	37825.9	32066.1	23354.6	21240.7	20796.6
	25%	24958.0	34772.6	29050.2	19638.0	19232.5	17666.3
	75%	35384.6	39912.6	33966.9	28679.9	23141.1	21938.7
	Bottom Whisker	18377.7	27825.7	21856.4	10454.5	18912.8	16872.2
	Top Whisker	52254.1	46007.6	42823.1	37807.2	25357.4	25586.4
	n	33	25	25	33	8	8
Particle Number Emission Rate / s^{-1}	Mean	8.2	120.9	490.8	64.3	195.0	479.6
	Median	9.0	145.0	624.9	60.1	202.3	480.7
	25%	3.4	45.8	229.6	44.2	163.9	435.0
	75%	22.6	285.1	1003.2	44.2	235.6	520.2
	Bottom Whisker	0.5	8.9	84.4	44.2	105.6	418.7
	Top Whisker	215.9	1153.2	3379.4	44.2	324.7	565.1
	n	33	25	25	33	8	8
Particle Mass Concentration / µg m^{-3}	Mean	0.026	0.135	0.376	0.485	1.008	2.919
	Median	0.024	0.172	0.422	0.390	1.201	2.851
	25%	0.011	0.072	0.240	0.266	0.735	2.016
	75%	0.068	0.344	0.656	0.954	1.490	4.606
	Bottom Whisker	0.001	0.006	0.034	0.104	0.363	1.339
	Top Whisker	0.285	1.085	1.843	9.081	1.692	5.613
	n	33	25	25	33	8	8
Particle Mass Emission Rate / ng s^{-1}	Mean	0.005	0.134	0.666	0.121	0.307	1.110
	Median	0.005	0.207	0.682	0.108	0.322	1.087
	25%	0.002	0.053	0.312	0.070	0.230	0.901
	75%	0.013	0.365	1.284	0.198	0.400	1.391
	Bottom Whisker	0.0001	0.004	0.032	0.030	0.123	0.627
	Top Whisker	0.063	1.151	4.209	1.311	0.725	1.983
	n	33	25	25	33	8	8
